# Auditory Time-Interval Perception as Causal Inference on Sound Sources

**DOI:** 10.3389/fpsyg.2012.00524

**Published:** 2012-11-28

**Authors:** Ken-ichi Sawai, Yoshiyuki Sato, Kazuyuki Aihara

**Affiliations:** ^1^Institute of Industrial Science, University of TokyoTokyo, Japan; ^2^Graduate School of Information Systems, University of Electro-CommunicationsTokyo, Japan

**Keywords:** time-interval perception, Bayesian inference, source identification, causal inference

## Abstract

Perception of a temporal pattern in a sub-second time scale is fundamental to conversation, music perception, and other kinds of sound communication. However, its mechanism is not fully understood. A simple example is hearing three successive sounds with short time intervals. The following misperception of the latter interval is known: underestimation of the latter interval when the former is a little shorter or much longer than the latter, and overestimation of the latter when the former is a little longer or much shorter than the latter. Although this misperception of auditory time intervals for simple stimuli might be a cue to understanding the mechanism of time-interval perception, there exists no model that comprehensively explains it. Considering a previous experiment demonstrating that illusory perception does not occur for stimulus sounds with different frequencies, it might be plausible to think that the underlying mechanism of time-interval perception involves a causal inference on sound sources: herein, different frequencies provide cues for different causes. We construct a Bayesian observer model of this time-interval perception. We introduce a probabilistic variable representing the causality of sounds in the model. As prior knowledge, the observer assumes that a single sound source produces periodic and short time intervals, which is consistent with several previous works. We conducted numerical simulations and confirmed that our model can reproduce the misperception of auditory time intervals. A similar phenomenon has also been reported in visual and tactile modalities, though the time ranges for these are wider. This suggests the existence of a common mechanism for temporal pattern perception over modalities. This is because these different properties can be interpreted as a difference in time resolutions, given that the time resolutions for vision and touch are lower than those for audition.

## 1 Introduction

Temporal pattern processing is necessary for all sensory modalities and these patterns contain much essential information for our brain to learn what happens in the external world. Therefore, revealing the temporal perception system is fundamental to understanding the sensory processing system, but it is not fully understood yet.

**Figure 1:**
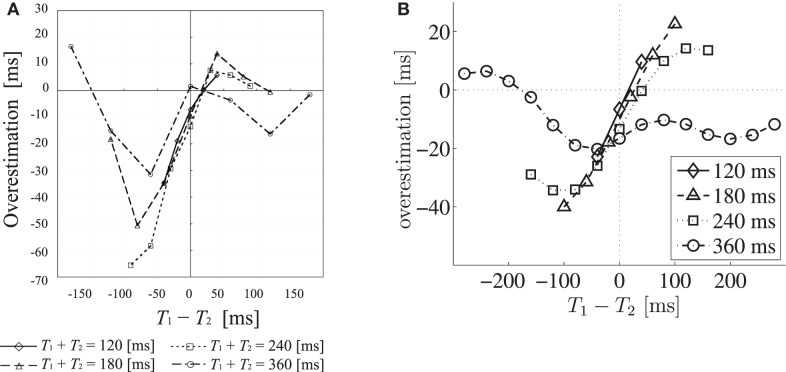
**Perceptual and simulated overestimation of *T*_2_ as a function of *T*_1_ − *T*_2_**. Each marker represents a different total duration *T*_1_ + *T*_2_. **(A)** Perceptual overestimation of *T*_2_. Perceptual overestimation is measured by using the method of adjustment. (From Figure 3B, Miyauchi and Nakajima, 2005, with changes in notations. ©University of California Press Journals. Adapted with permission.) **(B)** Simulated overestimation of *T*_2_, calculated by subtracting *T*_2_ from the expectation value of P(*T*_2_|*s*_1_,*s*_2_,*s*_3_). The area under the horizontal dashed line indicates the underestimation of *T*_2_, and the area on the left side of the vertical dashed line indicates *T*_1_ < *T*_2_.

Hearing three rapid successive sounds is a good situation for investigating the time-perception system. One reason for this is that the temporal accuracy of our auditory system is higher than those for other modalities (Burr et al., 2009; Vroomen and Keetels, 2010; Occelli et al., 2011); that is, auditory experimental results reflect the actual time-perception mechanism better. In addition, a combination of two time intervals is the simplest situation of temporal pattern perception. With regard to hearing three rapid sounds on a hundred-millisecond scale, it is known that our brain sometimes misestimates the second interval depending on the relative length of the two intervals. Concretely speaking, the second interval, *T*_2_, is perceived as shorter than the actual length in the case where *T*_2_ is equal to or a little longer than the first interval, *T*_1_. This perceptual underestimation phenomenon was named “time-shrinking” (Nakajima et al., 1991). This illusion vanishes as the total length *T*_1_ + *T*_2_ increases. In addition, though the degrees of misestimation are not so large as those for the case of the time-shrinking illusion, the following phenomena on the perception of *T*_2_ have also been observed (Miyauchi and Nakajima, 2005; Figure 1A): overestimation of *T*_2_ when *T*_2_ is a little shorter than *T*_1_; underestimation of *T*_2_ when *T*_2_ is much shorter than *T*_1_; and overestimation of *T*_2_ when *T*_2_ is much longer than *T*_1_. The time-shrinking illusion has been examined in other articles as well (Nakajima et al., 1992; ten Hoopen et al., 1993, 2006; Suetomi and Nakajima, 1998; Miyauchi and Nakajima, 2007; Mitsudo et al., 2009). Furthermore, it was reported that this phenomenon occurs in other sensory modalities such as visual (Arao et al., 2000) and tactile (van Erp and Spapé, 2008) senses. This fact suggests that there is a common time-perception system among sensory modalities.

A time-perception model has been proposed to explain the time-shrinking illusion (Nakajima et al., 2004). In this model, it is assumed that the subjective duration of a time-interval is proportional to the sum of the actual length and a constant length. It is also assumed that if the neural system judges the two neighboring intervals as similar, the estimating process for the latter interval is shortened and the latter interval is thus underestimated. By these assumptions, this model can quantitatively mimic the time-shrinking illusion, namely, the underestimation of *T*_2_ caused by a shorter preceding interval *T*_1_. However, the other misestimation phenomena when hearing three successive sounds are out of the scope of this model and cannot be reproduced by the model.

In the present study, we consider that the perceptual phenomena as mentioned above are results of effective information processing in our neural system. Sensory information, which our brain uses to infer what happens in the world, inevitably has uncertainty caused by both internal noise in our nervous system (Faisal et al., 2008) and ubiquitous fluctuation in the external world. Therefore, our brain must manage with those kinds of uncertainty, otherwise we may misunderstand the situation or regard the same experiences as different. One reasonable way for the brain to cope with the uncertainty is exploiting prior knowledge, or the experience and statistics pertaining to the situation. This strategy can be formulated by using Bayesian inference. Bayesian modeling is a powerful method for describing the human perception mechanism and has been applied to visual temporal perception (Miyazaki et al., 2005; Jazayeri and Shadlen, 2010), and more widely to human perception (Vilares and Körding, 2011, for a recent review).

## 2 Materials and Methods

To consider the perceptual phenomena of hearing three rapid sounds, we assume a Bayesian observer who tries to solve a common source identification problem for each pair of two neighboring sounds. Further, prior to hearing, the observer assumes that sounds from the same source have short and equal intervals. The assumption of prior knowledge of short time intervals for stimuli from the same source is based on some previous works. These studies showed that the closer the two sources, the shorter are the perceived time intervals (Akerboom et al., 1983, for audition; and Goldreich, 2007; Kuroki et al., 2010, for tactile sensation). Further, with respect to the assumption of equal intervals, we can find many examples of signals aligned at almost equal intervals: heart beats, swinging pendulum, etc. This can be because simple dynamical systems tend to generate periodical orbits, which are often observed as periodic signals generated by a limit cycle.

Here, we propose that the perception of sound intervals involves inference of causal relationship among sounds. Although there is little direct evidence for this notion, some auditory perceptual phenomenon could be associated with some form of causal judgments. For example, the time-shrinking illusion vanishes in the case wherein the temporal pattern is marked by sounds with quite different frequencies (Remijn et al., 1999). For this case, we consider that sounds with different frequencies have been judged as from independent sources. Therefore, the perceptual estimation of the latter time-interval is different from that for the case of a sound sequence composed of the same frequency. This view that sound frequency indicates source identity is also supported by an auditory psychological phenomenon (Deutsch, 1975). The perception of a common source is a kind of causal inference and should be important for making an effective inference (Körding et al., 2007; Sato et al., 2007; Shams and Beierholm, 2010). We will discuss this point further in Discussion.

**Figure 2:**
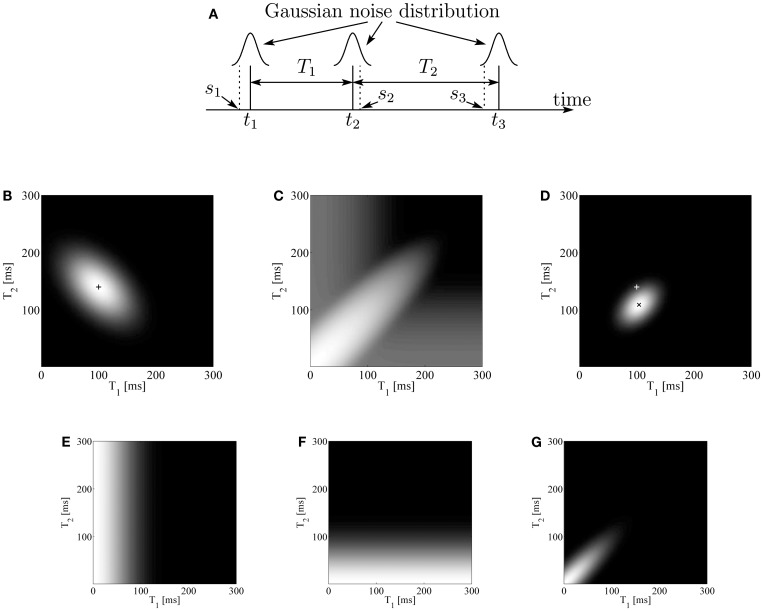
**(A)** A temporal pattern and its observation. The horizontal axis is time. Each solid vertical line indicates an actual sound timing *t_i_*, and each dashed vertical line indicates its observed timing *s_i_*. Each observation is made on the basis of an independent identical distribution. **(B)** Likelihood function of (*T*_1_,*T*_2_) given observed values of *S*_1_ = 100 ms and *S*_2_ = 140 ms (indicated as “+”). Intensity indicates the degree of likelihood. **(C)** Prior distribution of (*T*_1_,*T*_2_) with intensity on a logarithmic scale of the probability, illustrating that the prior takes a high value near both axes as well as along the 45° line from the *T*_1_-axis. **(D)** Posterior distribution of (*T*_1_,*T*_2_) given observed values of *S*_1_ = 100 ms and *S*_2_ = 140 ms (indicated as “+”). Intensity indicates probability. The cross sign (×) corresponds to the peak of the distribution. **(E–G)** Prior distributions of (*T*_1_,*T*_2_) given that **(E)** the first and second sounds come from the same source, **(F)** the second and third sounds come from the same source, and **(G)** all three sounds come from the same source. Intensity indicates probability.

Our Bayesian model assumes that our neural system cannot observe true time instants *t*_1_, *t*_2_, and *t*_3_ of the sounds, but only observed times including noise *s*_1_, *s*_2_, and *s*_3_, respectively (Figure 2A). Each index of the variables indicates the order of emergence in the sound sequence. Then, our brain infers true interval durations *T*_1_ = *t*_2_ − *t*_1_ and *T*_2_ = *t*_3_ − *t*_2_ from the observation. To estimate them, our Bayesian observer composes a conditional probability, called a posterior probability, P(*T*_1_,*T*_2_|*s*_1_,*s*_2_,*s*_3_). Bayesian theorem enables us to represent the posterior probability as

(1) $$\text{P}\left(T_{1},T_{2}\left|s_{1},s_{2},s_{3}\right.\right)=\frac{\text{P}\left(s_{1},s_{2},s_{3}\left|T_{1},T_{2}\right.\right)\text{P}\left(T_{1},T_{2}\right)}{\text{P}\left(s_{1},s_{2},s_{3}\right)}.$$

Since the denominator on the right side can be obtained by integrating the numerator over *T*_1_ and *T*_2_, we need to consider only terms P(*s*_1_,*s*_2_,*s*_3_|*T*_1_,*T*_2_) and P(*T*_1_,*T*_2_) in the numerator. The first term of the numerator represents how the observational values are obtained, and is formulated as

$$\begin{array}{llll} \text{P}\left(s_{1},s_{2},s_{3}\left|T_{1},T_{2}\right.\right) & =\int \text{P}\left(s_{1},s_{2},s_{3}\left|T_{1},T_{2},t_{2}\right.\right)\text{P}\left(t_{2}\right)\text{d}t_{2} & & \\ & \propto \int \text{P}\left(s_{1},s_{2},s_{3}\left|t_{1},t_{2},t_{3}\right.\right)\text{d}t_{2} & & \\ & =\int \text{P}\left(s_{1}\left|t_{1}\right.\right)\text{P}\left(s_{2}\left|t_{2}\right.\right)\text{P}\left(s_{3}\left|t_{3}\right.\right)\text{d}t_{2}, & & \text{(2)} \end{array}$$

where we assume that distribution P(*t*_2_) is constant; knowing *T*_1_, *T*_2_, and *t*_2_ is equivalent to knowing *t*_1_, *t*_2_, and *t*_3_ in the second line, and the noise distributions for the timings of the three sounds are assumed to be independent from each other in the third line. We set the distribution of the observation noise as a Gaussian distribution with the width σ*_o_* and the center at a true value given as

(3) $$\text{P}\left(s_{i}\left|t_{i}\right.\right)=\frac{1}{\sqrt{2\pi }\sigma_{o}}\exp\left(-\frac{{\left(s_{i}-t_{i}\right)}^{2}}{2\sigma_{o}^{2}}\right),\;\;\text{for}\;i=1,2,3.$$

Here, we consider standard deviation σ*_o_* to be constant with time. By substituting equation (3) into equation (2) and integrating over *t*_2_, we obtain the following formula (see Appendix for the details of this derivation):

$$\begin{array}{llll} & \text{P}\left(s_{1},s_{2},s_{3}\left|T_{1},T_{2}\right.\right) & & \\ & \;\propto \exp\left(-\frac{{\left(T_{1}-S_{1}\right)}^{2}+{\left(T_{2}-S_{2}\right)}^{2}+\left(T_{1}-S_{1}\right)\left(T_{2}-S_{2}\right)}{3\sigma_{o}^{2}}\right), & & \text{(4)} \end{array}$$

where we introduce variables *S*_1_ = *s*_2_ − *s*_1_ and *S*_2_ = *s*_3_ − *s*_2_, which represent the observed interval durations. Note that, given *T*_1_ and *T*_2_, *t*_1_ and *t*_3_ are not independent from *t*_2_ but change with *t*_2_. Therefore, the integral range of *t*_2_ in equation (2) is (−∞, ∞). This term stands for the likelihood of the true intervals. Due to (*T*_1_ − *S*_1_)(*T*_2_ − *S*_2_), this function has a negative correlation between *T*_1_ and *T*_2_, as shown in Figure 2B.

Then, we formulate term P(*T*_1_,*T*_2_) in equation (1). This term does not relate to *s*_i_(*i* = 1, 2, 3); that is, what our neural system has observed. Thus, this probability function represents knowledge acquired prior to the event. We model the prior knowledge of two neighboring time intervals as follows, assuming that the observer solves a source identification problem. First, our brain infers from the three successive sounds whether each pair of two neighboring sounds comes from the same source. To consider the source identification inference, we introduce variable *C* that represents which of the three sounds are from the same source. Here, our brain is not considered to make a judgment that the first and third sounds come from the same source while at the same time the second sound comes from another source. Thus, *C* represents the following four cases:

1. each sound is from an independent source,
2. the first and second sounds come from the same source and the third from another source,
3. the second and third sounds come from the same source and the first from another source,
4. all three sounds are from the same source.

Then, we assign 1, 2, 3, and 4 as the value of *C* to the above cases, respectively. Using the variable *C*, we formulate the prior distribution as

$$\begin{array}{llll} \text{P}\left(T_{1},T_{2}\right) & =\overset{4}{\underset{C=1}{\sum }}\;\text{P}\left(T_{1},T_{2},C\right) & & \\ & =\overset{4}{\underset{C=1}{\sum }}\;\text{P}\left(C\right)\text{P}\left(T_{1},T_{2}\left|C\right.\right). & & \text{(5)} \end{array}$$

We treat the probabilities of *C* appearing in equation (5) as model parameters, and denote P(*C* = *j*)(*j* = 1, 2, 3, 4) by *p_j_*.

Next, we formulate prior distributions P(*T*_1_,*T*_2_|*C*) for *C* = 1, 2, 3, and 4, by using the assumption of equal and short intervals for sounds from the same source. The assumption is formulated as follows:

- For *C* = 1, there is no bias for the sound intervals. Thus, the prior distribution is a two-dimensional uniform distribution:
  (6) $$\text{P}\left(T_{1},T_{2}\left|C=1\right.\right)=\frac{1}{L^{2}},$$
  where *L* is a parameter defining the integration range.
- For *C* = 2 and *C* = 3, the two sounds that come from the same source are expected to have a short interval (Figures 2E,F). Each prior distribution is as follows:
  $$\begin{array}{llll} \text{P}\left(T_{1},T_{2}\left|C=2\right.\right) & =\text{P}\left(T_{1}\left|C=2\right.\right)\text{P}\left(T_{2}\left|C=2\right.\right) & & \\ & =\frac{1}{\sqrt{2\pi }\sigma_{p}}\exp\left(-\frac{T_{1}^{2}}{2\sigma_{p}^{2}}\right)\cdot \frac{1}{L}, & & \text{(7)}\\ \text{P}\left(T_{1},T_{2}\left|C=3\right.\right) & =\frac{1}{L}\cdot \frac{1}{\sqrt{2\pi }\sigma_{p}}\exp\left(-\frac{T_{2}^{2}}{2\sigma_{p}^{2}}\right), & & \text{(8)} \end{array}$$
  where standard deviation σ*_p_* is a parameter that controls the bias toward short intervals. P(*T*_1_|*C* = 2) gives the distribution of an interval wherein the two marker sounds are from the same source, and P(*T*_2_|*C* = 2) gives the distribution of an interval wherein the two sounds come from different sources.
- For *C* = 4, the three markers are expected to have short and equal intervals. This distribution is expressed as a two-dimensional Gaussian distribution, with the center at the origin and a positive correlation between the two variables *T*_1_ and *T*_2_ (Figure 2G). Thus, this distribution can be expressed as
  (9) $$\text{P}\left(T_{1},T_{2}\left|C=4\right.\right)=\frac{1}{Z}\exp\left[-\left(\frac{{\left(T_{1}+T_{2}\right)}^{2}}{2\sigma_{q}^{2}}+\frac{{\left(T_{1}-T_{2}\right)}^{2}}{2\sigma_{r}^{2}}\right)\right],$$
  where *Z* is the normalization term, and σ*_q_* and σ*_r_* are constant parameters. It is necessary for the prior distribution to satisfy the following condition:
  (10) $$\int \text{P}\left(T_{1},T_{2}\left|C=4\right.\right)\text{d}T_{2}=\text{P}\left(T_{1}\left|C=2\right.\right).$$
  Given this condition, the constants *Z*, σ*_q_*, and σ*_r_* in equation (9) are represented as follows:
  $$\begin{array}{llll} & \sigma_{q}^{2}+\sigma_{r}^{2}=4\sigma_{p}^{2}, & & \text{(11)}\\ & Z=\pi \sigma_{q}\sigma_{r}. & & \text{(12)} \end{array}$$

New parameters σ*_q_* and σ*_r_* control the shape of the distribution. Since we intend the distribution to have a positive correlation between *T*_1_ and *T*_2_, σ*_q_* should be greater than σ*_r_*.

By substituting equations (6)–(9) into equation (5), we have prior distribution P(*T*_1_,*T*_2_). The obtained prior distribution has a large peak at the origin of the *T*_1_ − *T*_2_ plane, and also has high values along the *T*_1_ and *T*_2_ axes, and along the 45° line from the *T*_1_-axis (Figure 2C).

Then, we obtain the posterior distribution P(*T*_1_,*T*_2_|*s*_1_,*s*_2_,*s*_3_) by multiplying the likelihood function of equation (4) and the prior distribution (Figure 2D).

## 3 Result

We conducted a numerical simulation to show the validity of our model. The parameter values used in the simulation are shown in Table 1. There are too many parameters in our model to learn the correct values from appropriate experiments. Thus, the parameter values are chosen and adjusted so that the time scales are not strange in terms of their physical implications. For example, because the time resolution of the auditory system changes with measurement methods, a specific time resolution parameter σ*_o_* cannot be decided. Therefore, we set it so that the time scale is similar to existing psychological results (Grondin and Plourde, 2007, for example). The parameter value of *L* is decided so as to cover the time range in which the stimuli are presented.

**Table 1:** **Parameter values in the simulation**.

Parameter	Value	Description
σ*_o_*	25 ms	Time resolution of auditory system. The smaller this value is, the smaller is the perceptual shift
σ*_p_*	50 ms	Strength of the bias toward short intervals. The smaller this value is, the stronger is the bias and the larger is the perceptual shift
σ*_q_*, σ*_r_*)	(97.5, 22.2 ms)	Strength of the bias toward equal intervals. The larger the value of σ*_q_* is, the stronger is the bias and the larger is the perceptual shift for the case that the two intervals are similar
(*p*_1_, *p*_2_, *p*_3_, *p*_4_)	(0.01, 0.01, 0.01, 0.97)	Probability distribution of *C*. The larger the value of *p*_1_ is, the smaller is the perceptual shift. Increasing *p*_2_ and *p*_3_ results in a larger perceptual shift for the case that the two intervals are dissimilar. Increasing *p*_4_ results in a larger perceptual shift for the case that the two intervals are similar
*L*	500 ms	Integration range. The shorter this value is, the smaller is the perceptual shift especially for the case that the two intervals are similar

In this simulation, we calculated the expectation value of the marginal distribution of *T*_2_ and regarded the value as a result of the Bayesian observer’s inference. Although there are some other decision-making strategies, such as maximizing the posterior probability, we chose calculating the expectation value because of its low computational cost. However, the simulation result of the maximum *a posteriori* strategy was not qualitatively different from that of the expectation value. In addition, it is yet to be ascertained which rule should be applied to a Bayesian inference (see Jazayeri and Shadlen, 2010, for this issue).

Using this simulation, our model reproduced the time-shrinking illusion; that is, the large underestimation of *T*_2_ when *T*_2_ is a little longer than *T*_1_, due to the assumption of equal intervals. However, the amount of overestimation when *T*_2_ is a little shorter than *T*_1_ was smaller than the above underestimation. We also observed overestimation and underestimation of *T*_2_ when *T*_2_ is much longer and shorter than *T*_1_, respectively. Moreover, our model simulation showed that the underestimation and overestimation decrease as the total length increases and that there is underestimation of *T*_2_ when *T*_2_ = *T*_1_ (Figure 1B). These properties of our model were also observed in psychological experiments (Figure 1A).

### 3.1 Explanation of the perception of three rapid sounds

**Figure 3:**
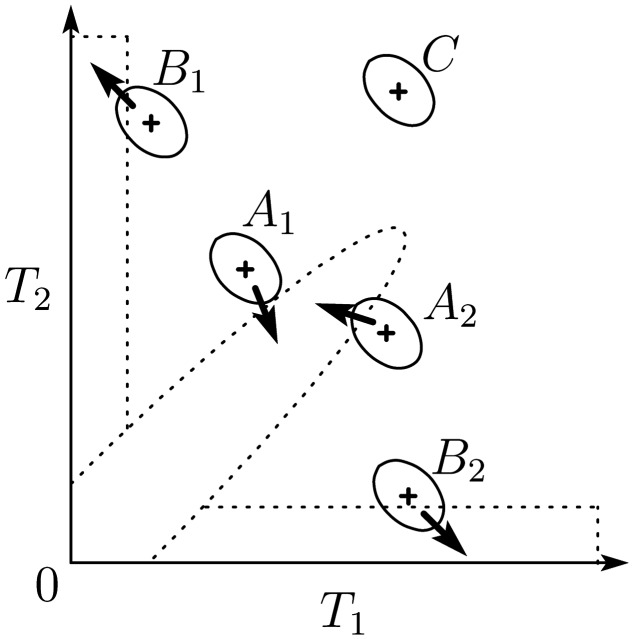
**Schematic figure of the model mechanism**. Dashed lines indicate the shape of the prior distribution. Each solid-lined ellipse represents the likelihood function given an observed interval pair marked as a plus sign on the center of the ellipse. Each arrow describes the direction of the perceptual shift.

Here, we explain how our model reproduces the behavior of the human auditory system. First, when the two time intervals are similar, the observed time-interval pair stands near the diagonal line on the *T*_1_-*T*_2_ plane. Thus, the perception of three sounds shifts from noisy observation toward prior knowledge when all three sounds originate from the same source. As a result, the two intervals are perceived as more similar to each other than their observation. That is, *T*_2_ is underestimated if *T*_2_ is a little longer than *T*_1_ (Point *A*_1_ in Figure 3), and *T*_2_ is overestimated if *T*_2_ is a little shorter than *T*_1_ (Point *A*_2_ in Figure 3). In addition, the degree of underestimation is larger than that of overestimation because the peak of the prior distribution is at the origin due to the expectation of short intervals. The expectation of short intervals also causes the underestimation of *T*_2_ when *T*_2_ = *T*_1_.

Next, when the intervals are dissimilar, the time-interval pair is located either near the *T*_1_-axis or the *T*_2_-axis on the *T*_1_-*T*_2_ plane. Therefore, perception is biased toward the *T*_1_-axis or the *T*_2_-axis by prior knowledge when the first two or the latter two sounds come from the same source, respectively. In addition, since the likelihood function has a negative correlation between *T*_1_ and *T*_2_, perception shifts along the negative correlation. Thus, *T*_2_ is perceived as longer than the actual duration if *T*_2_ is longer than *T*_1_, and vice versa (Points *B*_1_ and *B*_2_ in Figure 3, respectively).

In addition, the shape of the prior distribution becomes more flat as distance from the origin and the axes on the *T*_1_-*T*_2_ plane increases. Therefore, the prior effect is weak in such areas (Point *C* in Figure 3).

## Discussion

Our model succeeds in replicating the human perception of a simple temporal pattern. This result suggests that our brain judges the causality of sounds and expects short and equal intervals for temporal patterns in the unconscious process.

In our model, we assumed that the observer inferred the causal relationship among sounds. Although there is little evidence for this assumption, we can propose some experiments that could verify it. For example, we propose an experiment in which subjects hear three rapid sounds and report which of the three sounds come from the same source. The rate of each judgment on source identification can be predicted by calculating P(estimated *C|T*_1_,*T*_2_) from the present model. In addition, this experiment would also provide feedback on the parameter values of (*p*_1_, *p*_2_, *p*_3_, *p*_4_), which are rather arbitrary in this study. By extending our model, we can also predict that the temporal pattern of sounds alters the perception of their spatial locations. Although we modeled the perception of time intervals marked by sounds in this article, we can also model the spatial perception of the sounds in almost the same form of causal inference and easily combine it with the current model. From this combined model, we predict that the same spatial patterns of sounds are perceived as spatially different if the patterns are temporally different. This is because the inference on the causal relationship among sounds is made from their temporal and spatial pattern in this model, and thus varies with temporal difference even if the actual spatial patterns are the same.

Our model has several parameters, and there exists some arbitrariness in their setting. For instance, even if we change the value of *L* from that in Table 1 to another value, we can reproduce a result similar to Figure 1B by adjusting parameters (*p*_1_, …, *p*_4_). In this article, we choose quite a high value for *p*_4_ relative to the other three parameters. Although we assumed that inference was made based on observed time of sounds, in reality, we observe other features of sounds such as direction, pitch, color, volume, and so on, and all of these provide cues for the causal relationship among sounds. In the experiment we reproduced, all of these other features were kept the same for the series of three sounds, which strongly suggests that the sounds had come from the same source. We interpret (*p*_1_, …, *p*_4_) as including the cues from those other features. Thus, it might be natural to assume that *p*_4_, which is the probability of all of the sounds coming from the same source, is considerably higher than the other possibilities. This suggests that time-interval perception depends on other sound features and, if presented with visual stimuli, also depends on visual features such as color, size, or location. In fact, it was confirmed that the result of time-interval perception differs according to the combination of stimulus pitches (Remijn et al., 1999).

Our model could be improved by trying to replicate the experimental facts about the perception of *T*_1_. It was reported that the direction of the perceptual shift of *T*_1_ follows the same pattern as that of *T*_2_; that is, *T*_1_ is underestimated when *T*_1_ is a little longer or much shorter than *T*_2_, and *T*_1_ is overestimated when *T*_1_ is a little shorter or much longer than *T*_2_ (Miyauchi and Nakajima, 2005). This qualitative property of *T*_1_ perception can be predicted by our model. However, in that experiment, the magnitude of each perceptual shift of *T*_1_ was found to be less than that of *T*_2_. Since the present model has symmetry between *T*_1_ and *T*_2_, it is impossible for our model to mimic the difference between the perceptions of *T*_1_ and that of *T*_2_. In the future, we seek to consider how we refine the present model to reproduce experimental results on the perception of *T*_1_.

In auditory science, the issue is discerning a single sound stream in a complex of multiple sounds. This ability of the auditory system is called “auditory scene analysis” or “auditory scene segregation” (Bregman, 1990), and regarded as an important key to reveal the auditory system. Because this sound separating mechanism should involve perceptual source identification, our model may contribute to considering a sound segregation mechanism from the temporal aspect.

Finally, let us consider the time-perception mechanisms for other sensory modalities. From the psychological experiments on the visual (Arao et al., 2000) and tactile (van Erp and Spapé, 2008) time-shrinking illusions, it is known that time ranges for these modalities are broader than those for audition. The underlying reason can be understood by using the present model as follows, given that the visual and tactile time resolutions are lower than the auditory one. The perceptual bias of our model becomes weaker in a longer time scale. However, for a low-temporal-resolution modality, the perceptual bias is still relatively strong, because the observation has much uncertainty. Thus, the illusion occurs in a wider range. Though we can give a possible explanation for the difference among the modalities, the time-perception mechanisms in the sub-second scale for the other sensory modalities have not been well studied. Therefore, more research is needed before concluding that a time-perception system is shared by all sensory modalities.

## Conflict of Interest Statement

The authors declare that the research was conducted in the absence of any commercial or financial relationships that could be construed as a potential conflict of interest.

## Appendix

In this Appendix, we derive equation (4) from equations (2) and (3). First, by substituting equation (3) into equation (2), the likelihood function is written as

$$\begin{array}{llll} \text{P}\left(s_{1},s_{2},s_{3}\left|T_{1},T_{2}\right.\right) & \propto \overset{\infty }{\underset{-\infty }{\int }}\text{P}\left(s_{1}\left|t_{1}\right.\right)\text{P}\left(s_{2}\left|t_{2}\right.\right)\text{P}\left(s_{3}\left|t_{3}\right.\right)\text{d}t_{2}\; & & \\ & =\overset{\infty }{\underset{-\infty }{\int }}{\left(\frac{1}{\sqrt{2\pi }\sigma_{o}}\right)}^{3}\exp\left\{-\frac{1}{2\sigma_{o}^{2}}\left[{\left(s_{1}-t_{1}\right)}^{2}+{\left(s_{2}-t_{2}\right)}^{2}+{\left(s_{3}-t_{3}\right)}^{2}\right]\right\}\text{d}t_{2}\; & & \\ & \propto \overset{\infty }{\underset{-\infty }{\int }}\exp\left\{-\frac{1}{2\sigma_{o}^{2}}\left[{\left(s_{1}-t_{2}+T_{1}\right)}^{2}+{\left(s_{2}-t_{2}\right)}^{2}+{\left(s_{3}-t_{2}-T_{2}\right)}^{2}\right]\right\}\text{d}t_{2}. & & \text{(A1)} \end{array}$$

Then, by introducing variables *S*_1_ = *s*_2_ − *s*_1_ and *S*_2_ = *s*_3_ − *s*_2_, and substituting *u* for *t*_2_ − *s*_2_, we obtain equation (4) as follows:

$$\begin{array}{ll} \text{P}\left(s_{1},s_{2},s_{3}|T_{1},T_{2}\right)\alpha \int_{-\infty }^{\infty }\exp\left(-\frac{1}{2\sigma_{o}^{2}}\left\{{\left[\left(s_{2}-S_{1}\right)-t_{2}+T_{1}\right]}^{2}+{\left(s_{2}-t_{2}\right)}^{2}+{\left[\left(s_{2}+S_{2}\right)-t_{2}-T_{2}\right]}^{2}\right\}\right)\text{d}t_{\text{2}}\\ \;=\int_{-\infty }^{\infty }\exp\left\{-\frac{1}{2\sigma_{o}^{2}}\left[{\left(u-T_{1}+S_{1}\right)}^{2}+u^{2}+{\left(u+T_{2}-S_{2}\right)}^{2}\right]\right\}\text{d}u\\ \;=\int_{-\infty }^{\infty }\exp\left\{-\frac{1}{2\sigma_{o}^{2}}\left[3u^{2}-2\left(T_{1}-T_{2}-S_{1}+S_{2}\right)u+{\left(T_{1}-S_{1}\right)}^{2}+{\left(T_{2}-S_{2}\right)}^{2}\right]\right\}\text{d}u\\ \;=\int_{-\infty }^{\infty }\exp\left\{-\frac{1}{2\sigma_{o}^{2}}\left[3{\left(u-\frac{T_{1}-T_{2}-S_{1}+S_{2}}{3}\right)}^{2}\right]\right\}\text{d}u\\ \;\times \int_{-\infty }^{\infty }\exp\left\{-\frac{1}{2\sigma_{o}^{2}}\left[-\frac{{\left(T_{1}-T_{2}-S_{1}+S_{2}\right)}^{2}}{3}+{\left(T_{1}-S_{1}\right)}^{2}+{\left(T_{2}-S_{2}\right)}^{2}\right]\right\}\\ \;\alpha \exp\left[-\frac{{\left(T_{1}-S_{1}\right)}^{2}+{\left(T_{2}-S_{2}\right)}^{2}+\left(T_{1}-S_{1}\right)+\left(T_{2}-S_{2}\right)}{3\sigma_{o}^{2}}\right]. & \text{(A2)} \end{array}$$
